# Electrogenic CH_4_ oxidation on a bioanode: putative extracellular electron transport system in *Methylobacter* sp

**DOI:** 10.1093/femsec/fiag067

**Published:** 2026-06-23

**Authors:** Peter A G ter Horst, Ian P G Marshall, Reinier A Egas, Robin Klomp, Merijn A W Schutgens, Theo van Alen, Mike S M Jetten, Caroline P Slomp, Cornelia U Welte

**Affiliations:** Department of Microbiology, Radboud Institute for Biological and Environmental Sciences, Radboud University, Heyendaalseweg 135, 6525AJ Nijmegen, the Netherlands; Center for Electromicrobiology, Section for Microbiology, Department of Biology, Aarhus University, Ny Munkegade 114-116, 8000 Aarhus C, Denmark; Department of Microbiology, Radboud Institute for Biological and Environmental Sciences, Radboud University, Heyendaalseweg 135, 6525AJ Nijmegen, the Netherlands; Department of Microbiology, Radboud Institute for Biological and Environmental Sciences, Radboud University, Heyendaalseweg 135, 6525AJ Nijmegen, the Netherlands; Department of Microbiology, Radboud Institute for Biological and Environmental Sciences, Radboud University, Heyendaalseweg 135, 6525AJ Nijmegen, the Netherlands; Department of Microbiology, Radboud Institute for Biological and Environmental Sciences, Radboud University, Heyendaalseweg 135, 6525AJ Nijmegen, the Netherlands; Department of Microbiology, Radboud Institute for Biological and Environmental Sciences, Radboud University, Heyendaalseweg 135, 6525AJ Nijmegen, the Netherlands; Department of Microbiology, Radboud Institute for Biological and Environmental Sciences, Radboud University, Heyendaalseweg 135, 6525AJ Nijmegen, the Netherlands; Department of Microbiology, Radboud Institute for Biological and Environmental Sciences, Radboud University, Heyendaalseweg 135, 6525AJ Nijmegen, the Netherlands

**Keywords:** CH_4_ oxidation, aerobic methanotrophic bacteria, extracellular electron transfer, *Methylobacter*, multiheme c-type cytochrome

## Abstract

Aerobic methanotrophs are frequently detected in oxygen-limited, stratified coastal environments. Known adaptations, including high-affinity terminal oxidases and oxygen-binding bacteriohemerythrins, help explain methane oxidation at extremely low oxygen concentrations, yet their activity and ecological role under fully anoxic conditions remain uncertain. Here, we show that an anoxic, poised-anode bioelectrochemical system inoculated with a methane-oxidizing sediment enrichment produced methane-dependent current, with rapid current loss upon methane removal and recovery after re-addition. Metagenomic analysis revealed the selective enrichment of a *Methylobacter* population encoding a porin-cytochrome complex and numerous multiheme *c*-type cytochromes, suggesting extracellular electron transfer potential. A complementary phylogenomic survey across Methylococcales identified homologs of this gene cluster in multiple lineages, but with a scattered phylogenetic distribution indicative of modular acquisition. Comparative synteny further revealed conserved gene order across genomes, supporting horizontal transfer of the locus as a functional unit. Together, these results demonstrate that aerobic methanotrophs may employ extracellular electron transfer strategies to dissipate methane-derived electrons when oxygen-dependent respiration is constrained.

## Teaser

Diverse *Methylobacter* contain machinery for extracellular electron transport.

## Introduction

Methane (CH_4_) is a potent greenhouse gas and CH_4_ emissions from the coastal and open oceans are estimated at 4.8–28.4 Tg CH_4_ yr⁻¹, with continental shelf systems (<200 m water depth) contributing a very large share (3.6–20.4 Tg CH_4_ yr⁻¹; Rosentreter et al. 2021) due to high sedimentary production, ebullition and only partial removal (Bange et al. 2024).

The pathways supporting CH_4_ oxidation are governed by redox conditions and the availability of suitable terminal electron acceptors. Because of the relatively low redox potential of the CH_4_/CO₂ couple (E°′= −0.24 V), CH_4_ can be oxidized using a range of electron acceptors (such as sulfate, metal-oxides, humic acids, and nitrate) by consortia of methanotrophic archaea (ANME; Boetius et al. 2000, Beal et al. 2009, Knittel and Boetius 2009, Haroon et al. 2013, Scheller et al. 2016, Thauer 2011, Wallenius et al. 2021).

Besides ANME archaea, aerobic methanotrophic bacteria, particularly *Methylobacter* and *Methylomonas* spp. affiliated with Methylococcales, are frequently detected at oxic-anoxic interfaces, and even in anoxic zones in lakes and coastal systems. Incubation experiments demonstrate measurable CH_4_ oxidation by these bacteria at micromolar, nanomolar, and undetectable O₂ concentrations, indicating CH_4_ activation under extremely low or transient O₂ availability (Blees et al. 2014, Bar-Or et al. 2017, Martinez-Cruz et al. 2017, Mayr et al. 2020, Steinsdóttir et al. 2022, Venetz et al. 2023). High-resolution geochemical and genomic analyses suggest that environments classified as anoxic may contain microscale oxygen availability, providing a potential explanation for sustained aerobic methanotrophic activity under “anoxic” conditions (Ruff et al. 2024). Notably, *Methylobacter*-like methanotrophs sustain growth and CH_4_ oxidation in experiments with anoxic conditions designed to mimic hypolimnia (Schorn et al. 2024). In such redox-stratified environments, CH_4_ oxidation rates increased upon addition of Fe(III) oxides, Mn(IV) oxides, or humic analogs in incubations from oxycline waters, even when trace oxygen may still be required for initial CH_4_ activation (Oswald et al. 2016, Van Grinsven et al. 2021, Li et al. 2023, Vigderovich et al. 2023).

DNA-stable isotope probing further revealed direct assimilation of CH_4_-derived carbon by *Methylobacter* under strictly anoxic conditions, with methanotrophic activity modulated by electron acceptor availability, including Fe(III) minerals (Yang et al. 2025). Complementary hypoxic soil incubations demonstrated ferrihydrite-dependent CH_4_ oxidation and suggested involvement of *c*-type cytochromes and flavin-like redox mediators in electron transfer, although the mechanistic basis remains unresolved. These findings support the capacity of aerobic methanotrophic bacteria to utilize insoluble Fe(III) minerals under oxygen-limited conditions (Zheng et al. 2020, Yu et al. 2024).

Insoluble metal oxides represent extracellular electron acceptors, implying that CH_4_ oxidation coupled to metal reduction under anoxic conditions requires mechanisms for extracellular electron transfer (EET). Recent work showed that alphaproteobacterial methanotrophs could couple methane oxidation under oxygen-limited conditions to Fe(III) reduction via cytochrome-rich cell surface proteins and ferrihydrite-associated microtubular structures that facilitated electron transfer to Fe(III) minerals (Dai et al. 2025). Across electrogenic bacteria, outer-membrane multiheme cytochromes (MHCs) and porin-cytochrome complexes (PCC) are assumed to mediate EET to insoluble extracellular acceptors such as metal oxides and electrodes (Paquete et al. 2022). In *Shewanella oneidensis* MR-1, electrons flow from the quinol pool via CymA (inner-membrane tetraheme) to soluble, periplasmic *c*-type cytochrome and the decaheme *c*-type cytochrome MtrA within the outer-membrane β-barrel MtrB. These electrons are discharged to extracellular electron acceptors by outer-surface decahemes MtrC/OmcA (Bewley et al. 2012, Fonseca et al. 2013). Although such systems have not yet been reported for aerobic methanotrophs, pure cultures of *Methylococcus capsulatus* (Bath) and *Methylosinus trichosporium* OB3b generate power in CH_4_-fed microbial fuel cells, with cytochrome-mediated electron transfer implicated (Jawaharraj et al. 2021).

Collectively, these observations raise the possibility that EET may offload CH_4_-derived electrons from methane-oxidizing bacteria (MOB) to insoluble electron acceptors. We hypothesize that, under anoxic conditions, a CH_4_-dependent electrogenic biofilm can develop on a poised electrode in a bioelectrochemical system (BES). Here, we aimed to enrich electrogenic methanotrophic organisms from Bothnian Sea sediments and elucidate their potential EET pathways with electrochemical measurements and genome-resolved metagenomic analyses.

## Experimental procedures

### Site description, sampling, and inoculum preparation

A previously established CH_4_ + graphene oxide sediment enrichment culture from station US2, 16–20 cm below seafloor (cmbsf; Klomp et al. 2026) was used as inoculum. The US2 site context, sampling, incubation, analytical methods, porewater and solid-phase geochemistry, and microbial community characteristics are described elsewhere (Klomp et al. 2026). The enrichment bottle was stored at 4°C for 6 months, then re-acclimated by adding 0.5 bar CH_4_ to the headspace and incubated for 3 weeks in the dark at room temperature (20°C–22°C), while shaking at 100 RPM to deplete residual soluble electron acceptors before BES inoculation.

### BES operation and medium

A double-chamber BES was constructed with a RALEX® heterogeneous cation-exchange membrane (CMHPES; Mega, Prague, Czech Republic). The BES was operated with a MultiEmStat4 LR potentiostat (PalmSens, Houten, NL) using a 3 M Ag/AgCl reference electrode (Prosense, Oosterhout, NL) at a potential of +600 mV vs. SHE. The cathodes consisted of stainless-steel mesh (Goodfellow, Huntingdon, UK) and the anode of AvCarb G475A graphite felt (5.5 × 2.5 × 0.47 cm; Fuel Cell Store, Bryan, USA) mounted on a platinum wire (Goodfellow, Huntingdon, UK) as a current collector. Current was recorded every 60 s using MultiTrace software (PalmSens, Houten, NL) in chronoamperometric mode.

Under anoxic conditions, the anode chamber was inoculated with the aforementioned enrichment (originally containing 12 g of sediment in 36 ml of medium). Following inoculation, the BES was operated for an initial stabilization period of 10 days to allow for microbial attachment and biofilm establishment on the anode.

The anode chamber contained 300 ml artificial sulfate-free seawater medium containing per liter: 5.2 g NaCl, 1.0 g MgCl_2_ ∙ 2 H_2_O, 0.28 g CaCl_2_ ∙ 2 H_2_O, 0.1 g KCl, 53 mg NH_4_Cl, 34 mg KH_2_PO_4_, and was supplemented with 135 µl trace element solution, 100 µl vitamin solution, and 20 mM HEPES adjusted to pH 8.2. The trace element stock solution contained per liter: 1.35 g FeCl_2_ · 4 H_2_O, 100 mg MnCl_2_ · 4 H_2_O, 24 mg CoCl_2_ · 6 H_2_O, 100 mg CaCl_2_ · 2 H_2_O, 100 mg ZnCl_2_, 25 mg CuCl_2_ · 2 H_2_O, 10 mg H_3_BO_3_, 24 mg Na_2_MoO_4_ · 2 H_2_O, 220 mg NiCl_2_ · 6 H_2_O, 17 mg Na_2_SeO_3_, 4 mg Na_2_WO_4_ · 2 H_2_O, and 12.8 g nitrilotriacetic acid. The vitamin stock solution contained per liter 20 mg biotin, 20 mg folic acid, 100 mg pyridoxine-HCl, 50 mg thiamin-HCl · 2 H_2_O, 50 mg riboflavin, 50 mg nicotinic acid, 50 mg D-Ca-pantothenate, 2 mg vitamin B12, 50 mg p-aminobenzoic acid, and 50 mg lipoic acid. The cathode chamber contained 250 ml of the same medium, buffered with 100 mM HEPES adjusted to pH 8.2. The anolyte was stirred with a magnetic stirring bar at 320 RPM, whereas the catholyte was stirred at 230 RPM. Additionally, to suppress the growth of unwanted electrogenic heterotrophs, the anolyte was supplemented with a mixture of antibiotics with a final concentration of 50 ug/ml streptomycin sulfate, vancomycin HCl, ampicillin sodium salt, and kanamycin sulfate per antibiotic (Wissink et al. 2024). Every week, another 50 µg/ml of all antibiotics was supplemented to the BES. The anolyte was not replaced during the entire incubation period. The Ag/AgCl reference electrode was replaced after 40 days, correcting a 42.3 mV potential offset.

Gas feeds were continuously supplied: the anode with CH_4_/CO₂ (95%/5%) at 10 ml/min and N₂ (> 99%) at 2.2 ml/min, and the cathode with N₂ (> 99%) at 12.2 ml/min. The BES was incubated in the dark at room temperature for 45 days. During operation, pH of the anolyte and catholyte, and possible O₂ leakage into the anode, were monitored weekly. O₂ was measured with a Fibox 4 oxygen meter in combination with SP-PSt6-NAU oxygen trace spots (PreSens, Regensburg, Germany).

### DNA isolation and sequencing

DNA was extracted from two samples: the inoculum, obtained by centrifuging 2 ml of enrichment slurry at 10 000 × g for 10 min immediately before BES inoculation, and a quarter of the BES anode collected after 21 days of chronoamperometric operation (piece dimensions 1.4 × 0.6 × 0.47 cm). The electrode piece was flash-frozen in liquid N₂ and aseptically minced into ∼5 × 5 mm fragments, which were distributed across four PowerBead Pro tubes (DNeasy PowerSoil Pro Kit; Qiagen, Venlo, Netherlands). Extractions followed the manufacturer’s protocol with minor modifications: bead-beating 10 min at 50 Hz on a TissueLyser II (Qiagen, Venlo, Netherlands), pooling lysates from the four tubes and loading sequentially onto a single spin column, and eluting in 30 µl (instead of 50–100 µl). To inactivate residual nucleases, eluates were heated at 65°C for 10 min and stored at 4°C before sequencing.

A sequencing library was prepared with the Nextera XT DNA Library Preparation Kit (Illumina, San Diego, USA) according to the manufacturer’s instructions. Library quality and size distribution were assessed using the Agilent 2100 Bioanalyzer with the High Sensitivity DNA Kit (Agilent Technologies, Santa Clara, USA). Quantification of the library was performed with the Qubit dsDNA HS Assay Kit (Thermo Fisher Scientific, Waltham, USA). Paired-end sequencing (2 × 300 bp) was carried out on an Illumina MiSeq sequencer (Illumina, San Diego, USA) using the MiSeq Reagent Kit v3, following the manufacturer’s protocol.

### Metagenomic analysis

Metagenomic sequencing data were processed with Aviary v0.11.0 (Newell, Aroney and Zaugg 2024), using default settings. Reads were assembled with metaSPAdes (Nurk et al. 2017), while read-to-assembly alignment was performed using Minimap2 (Li 2018) and Samtools (Li et al. 2009), followed by coverage estimation via CoverM (Aroney et al. 2025). Genome binning was performed with CONCOCT (Alneberg et al. 2014), VAMB (Nissen et al. 2021), MetaBAT (Kang et al. 2015), MetaBAT2 (Yue et al. 2020), MaxBin2 (Wu et al. 2016), and Rosella (Newell et al. 2023). Resulting bins were subsequently integrated and dereplicated using DASTool (Sieber et al. 2018). Quality assessment of bins was conducted using CheckM2 (Chklovski et al. 2023), and only metagenome assembled genomes (MAGs) with >50% completeness and <10% contamination were retained. Taxonomic assignment utilized GTDB-Tk v2.4.1 (Chaumeil et al. 2022) against the GTDB reference database (226^th^ release; Parks et al. 2022).

Relative abundance of each retained MAG was estimated using coverage-normalized mapping statistics in CoverM and is reported as the fraction of total mapped community coverage attributed to each genome, with the remaining fraction reported as unmapped. In parallel, community composition was assessed using the marker gene-based approach implemented in SingleM v0.19.0 (Woodcroft et al. 2025) to provide a taxonomic profile independent of genome recovery. SingleM yielded a comparable fraction of unassigned reads, indicating that incomplete genome recovery rather than taxonomic misclassification accounted for most unmapped reads. Therefore, abundance estimates reported throughout this study are based on CoverM read mapping to retained MAGs. SingleM was additionally applied to the metagenome of the original in situ sediment (16–20 cmbsf) for comparison.

Retained MAGs were screened for CxxCH heme-binding motifs using a custom R script (Supplementary Methods 1), and local genomic contexts of MHC genes were visualized in Artemis v15 (Rutherford et al. 2000). Functional annotation and metabolic reconstruction were conducted with Prokka v1.13 (Seemann 2014), MetaScan v1.2-beta (Cremers et al. 2022), and KEGG Mapper Reconstruct (Kanehisa et al. 2022).

Putative extracellular EET pathway proteins were structurally modeled using AlphaFold 3.0 (Abramson et al. 2024). Structural predictions were complemented with signal peptide analysis via SignalP 6.0 (Teufel et al. 2022) and membrane topology prediction via DeepTMHMM v1.0 (Hallgren et al. 2022).

### Phylogenomic analysis

Species-representative genomes with ≥99% CheckM2 estimated completeness classified as Methylococcales were retrieved from the globDB database release 226 (Speth et al. 2025).

Homologs of the four target proteins, selected based on CxxCH motif screening and annotation of the Methylobacter_1 MAG, namely the periplasmic monoheme c-type cytochrome (Methylobacter_1_02045), the decaheme c-type cytochrome (Methylobacter_1_02046; MtrA family), the outer-membrane β-barrel porin (Methylobacter_1_02047; MtrB/PioB family), and the inner-membrane-anchored monoheme c-type cytochrome (Methylobacter_1_02048), were identified by BLASTp version 2.11.0+ (Camacho et al. 2009) searches against the selected genome set. Hits were retained if alignments covered ≥ 90% of the query protein length (query coverage) and showed ≥ 70% amino acid identity. In parallel, genomes were screened for iron metabolism-related genes using FeGenie version 1.0 (Garber et al. 2020).

A whole-genome phylogenetic tree was constructed using GTDB-Tk version 2.5.2 (Parks et al. 2022) and GTDB-Tk reference data version r220 to generate a concatenated alignment of 120 marker genes, followed by tree inference with FastTree version 2.2 (Price et al. 2010) using default settings (Jones-Taylor-Thorton+CAT model), no bootstrapping was applied. The resulting phylogeny was visualized and annotated using ETE3 version 3.1.3 (Huerta-Cepas et al. 2016), with BLAST and FeGenie hits mapped onto the tree.

### Gene cluster homology and synteny analysis

Synteny between homologous gene clusters was assessed and visualized using Clinker v0.0.32 (Gilchrist Chooi 2021). Genomic regions of interest were extracted from annotated genomes retrieved from the GlobDB release 226 (Speth et al. 2025) and merged with the obtained *Methylobacter* MAG. Genomes were selected based on the phylogenomic analysis and restricted to those encoding BLASTp-detected homologs of the complete gene cluster or near-complete clusters lacking only the periplasmic cytochrome *c* component. Protein-coding sequences were compared using all-versus-all BLASTp with default parameters. Homologous genes were identified based on sequence similarity and visualized as linked blocks. Gene cluster alignments and synteny plots were rendered using clustermap.js, with gene orientation and relative genomic positions preserved.

## Results

### CH_4_ oxidation by a Bothnian Sea sediment enrichment drives current generation in a BES

**Figure 1:**
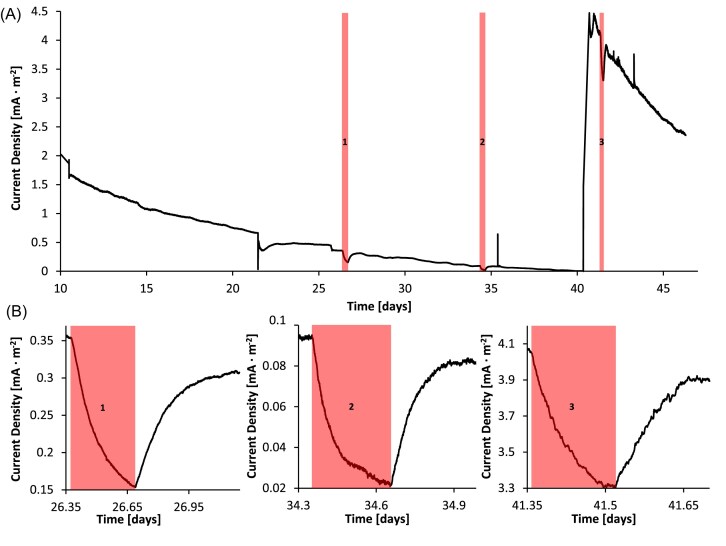
Current density during repeated CH_4_ removal experiments. (A) Current density over time with the anodic gas feed alternated between CH_4_/CO₂ and Ar/CO₂. Shaded windows indicate CH_4_ removal periods; numbers (1–3) denote the first, second, and third removal, respectively. (B) Corresponding magnified views of each removal period. The reference electrode was replaced after 40 days, correcting a 42.3 mV potential offset.

A bioelectrochemical system was inoculated with a CH_4_-oxidizing anaerobic microcosm enrichment from the Bothnian Sea to obtain CH_4_-oxidizing microorganisms capable of electrode-associated activity. During a 45-day incubation, the system produced a low but stable anodic current under continuous CH_4_ supply (Fig. 1A; Supplementary Data 1). Oxygen concentrations remained low throughout the experiment, with values below 0.08 µM after an initial transient peak of 1.12 µM (Supplementary Data 1).

To test whether current production depended on CH_4_ availability, the anodic gas feed was repeatedly switched from CH_4_/CO₂ to argon/CO₂. Each switch resulted in a rapid decrease in current during the CH_4_-off periods (shaded windows 1–3, Fig. 1), followed by recovery after CH_4_ reintroduction outside these intervals. In all three tests, current loss occurred shortly after CH_4_ removal, demonstrating a direct coupling between CH_4_ availability and anodic current production. The highest absolute CH_4_-dependent current density reached 0.6 mA m⁻² (test 3; Supplementary Data 1), while the highest relative CH_4_-dependent current accounted for 74.4% of total current at 61 µA m⁻² (test 2; Supplementary Data 1).

### Selective enrichment of *Methylobacter* spp. on the anode

**Figure 2:**
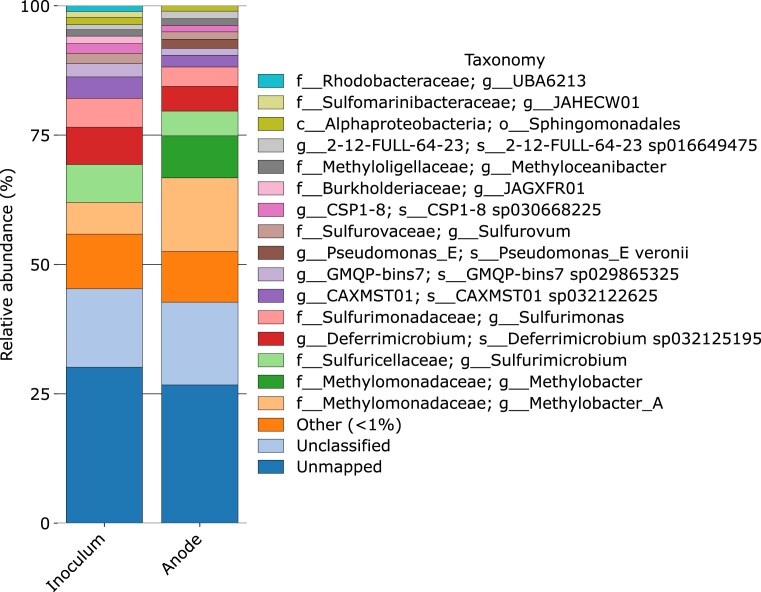
Genome-resolved community composition of the inoculum and anodic biofilm. Stacked bars show relative abundances of retrieved MAGs. Taxa are shown at the lowest resolved rank; bins lacking GTDB-Tk classification are grouped as “Unclassified,” reads not mapping to retained MAGs as “Unmapped,” and taxa <1% as “Other.” Biomass for DNA extraction of the anode community was harvested on operation day 21.

The microbial community composition of the inoculum and anodic biofilm was characterized by genome-resolved abundance profiling of retained MAGs using CoverM (Fig. 2; Supplementary Data 2).

Marker gene-based profiling with SingleM yielded comparable trends but did not substantially reduce the fraction of unassigned reads. According to the GTDB taxonomy, *Methylobacter* is subdivided into the canonical *Methylobacter* lineage (type species *Methylobacter luteus*) and three phylogenetically distinct clades (A–C). SingleM detected *Methylobacter* clade C only at trace relative abundance in the original sediment (Supplementary Data 3). The BES inoculum exhibited high taxonomic diversity and was dominated by *Sulfurimicrobium* (7.3%), *Deferrimicrobium* sp032125195 (7.2%), *Methylobacter* clade A (6.1%), *Sulfurimonas* (5.6%), CAXMST01 sp032122625 (Actinomycetota; 70–9 family; 4.1%), GMQP-bins7 (Gaiellaceae; 2.6%), and *Sulfurovum* (2.0%).

The operation of the BES resulted in pronounced shifts of the community on the anode. A *Methylobacter* MAG affiliated with the canonical lineage was below the detection limit in the inoculum but increased to 8.1% relative abundance in the anodic biofilm. In addition, a *Methylobacter* clade A MAG increased from 6.1% in the inoculum to 14.2% in the biofilm. In contrast, several taxa decreased in relative abundance on the anode, including *Deferrimicrobium* sp032125195 (7.2%–4.8%), *Sulfurimicrobium* (7.3%–4.8%), *Sulfurimonas* (5.6%–3.8%), and GMQP-bins7 (2.6%–1.3%). The fraction of unmapped reads remained substantial in both samples (30.1% in the inoculum and 26.7% in the anodic biofilm), indicating that part of the community was not captured by the genome-resolved read-mapping approach.

Together, the marked increase in both *Methylobacter* populations in the electrode-associated biofilm indicates selective enrichment of *Methylobacter* spp. under anode-associated bioelectrochemical conditions.

### The *Methylobacter* genome encodes pathways for CH_4_ oxidation, sulfur metabolism, and EET but not for nitrate reduction

**Figure 3:**
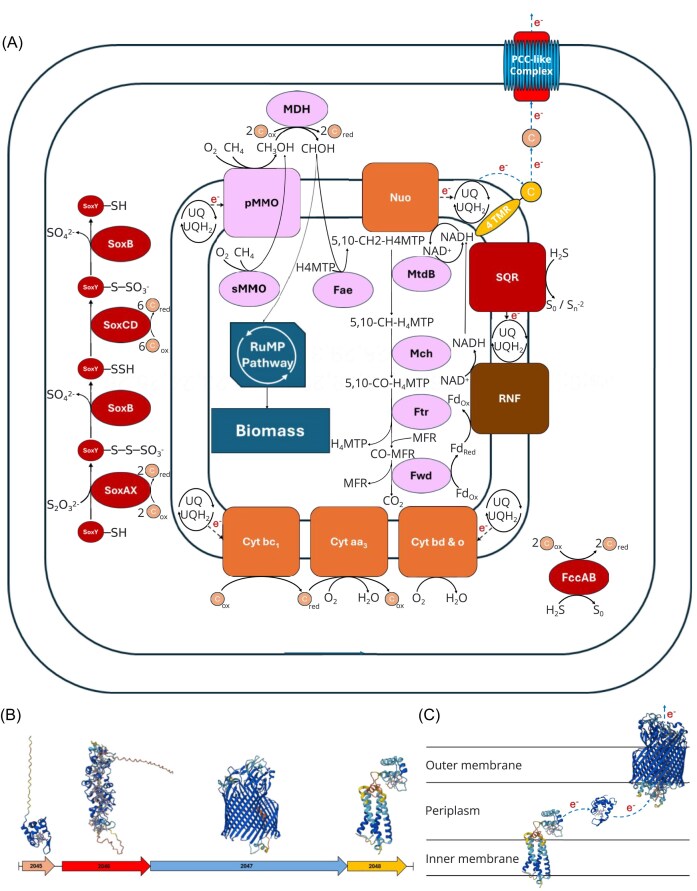
Metabolic reconstruction and predicted EET architecture of the *Methylobacter* MAG. (A) Schematic overview of pathways encoded by the genome, including methane oxidation by particulate and soluble methane mono-oxygenases (pMMO, sMMO) and methanol dehydrogenase (MDH). The particulate methane mono-oxygenase corresponds to a canonical *pmoCAB* gene cluster, and the soluble methane mono-oxygenase to *mmoXYZBDC*. Methanol dehydrogenases include both calcium-dependent Mxa-type (*mxaF*) and lanthanide-dependent XoxF-type (*xoxF*) enzymes. Formaldehyde oxidation and assimilation via the ribulose monophosphate (RuMP) pathway and C₁-processing enzymes (Fae, formaldehyde-activating enzyme; MtdB, methylene-tetrahydromethanopterin dehydrogenase; Mch, methenyl-H_4_MPT cyclohydrolase; Ftr, formyltransferase; Fwd, formylmethanofuran dehydrogenase). Sulfur metabolism comprises the Sox multienzyme complex (SoxABCDXY), sulfide: quinone oxidoreductase (SQR), and sulfide dehydrogenase (FccAB). The respiratory chain comprises NADH: quinone oxidoreductase (Nuo), cytochrome *bc₁*, cytochrome *aa₃* oxidase, and *bd*- and *o*-type quinol oxidases. The RNF complex functions as a ferredoxin: NAD⁺ oxidoreductase. “C” denotes *c*-type cytochromes, 4 TMR indicates the inner membrane-anchored cytochrome, and the outer membrane conduit is depicted as a PCC-like (porin-cytochrome complex). (B) Organization of a putative extracellular EET gene cluster (locus tags 2045–2048) with predicted protein structures including signal peptides. (C) Predicted trans-envelope architecture of the EET conduit. Protein structures of 2046–2047 were generated as a complex, whereas 2045 and 2048 were modeled separately; models were generated without signal peptides. Arrows indicate outward electron transfer toward extracellular acceptors.

The high-quality *Methylobacter* MAG (completeness 99.39%, contamination 3.92%; not affiliated with clade A) encoded the genetic potential for aerobic CH_4_ oxidation, thiosulfate and sulfide oxidation, and central carbon metabolism, including a complete tricarboxylic acid (TCA) cycle, the pentose phosphate pathway, and the Entner-Doudoroff pathway. In addition, the genome encoded a canonical aerobic respiratory chain with adaptations to low-oxygen conditions, as well as several proteins potentially involved in EET (Fig. 3A).

Canonical marker genes for CH_4_ oxidation were present, including genes encoding both particulate and soluble CH_4_ mono-oxygenases (*pmoCAB; mmoXYZBDC*). Genes encoding methanol dehydrogenases of both the calcium-dependent (*mxaF*) and lanthanide-dependent (*xoxF*) types were detected. Formaldehyde derived from methanol oxidation could be processed via two routes: complete oxidation to CO₂, supported by *fae, ftr, mtdB, mch*, and *fwdABCDE*, or assimilation into biomass via a complete ribulose monophosphate (RuMP) pathway (*hxlA, hps/phi, fae-hps, mcl*).

Beyond CH_4_ metabolism, the MAG encoded genes for sulfur compound oxidation, including the thiosulfate-oxidizing Sox multienzyme system (*soxXYZABCD*), sulfide: quinone oxidoreductase (*sqr*), and sulfide dehydrogenase (*fccAB*), indicating the capacity to oxidize reduced sulfur compounds. The genome further encoded an aerobic respiratory chain comprised of a cytochrome *bc₁* complex, a canonical cytochrome *aa₃* oxidase, and *bd*- and *o*-type quinol oxidases. Together with the presence of a bacteriohemerythrin, these features are indicative of respiratory potential across a broad range of oxygen concentrations.

Notably, genes encoding respiratory nitrate reduction (narGHI/napAB) were absent from the MAG, whereas genes for assimilatory nitrate and nitrite reduction (narB/nasAB, nasBDE, and nirBD) were present. This suggests that nitrate and nitrite can serve primarily as nitrogen sources for biosynthesis but are unlikely to function as terminal electron acceptors for energy conservation under anoxic conditions. Although the MAG encoded nitric oxide reductase (norBC), enabling potential reduction of NO to N₂O, this capacity likely reflects nitric oxide detoxification rather than a canonical denitrification pathway.

In this metabolic context, the *Methylobacter* MAG encoded a large repertoire of proteins associated with electron transfer processes. The MAG encoded 74 proteins containing at least one CxxCH motif encoding *c*-type cytochromes. Six of these were multiheme *c*-type cytochromes (MHCs; ≥2 CxxCH motifs), including a decaheme *c*-type cytochrome belonging to the DmsE/MtrA family. This decaheme *c*-type cytochrome was located within a gene cluster (Methylobacter_1_02045-Methylobacter_1_02048; Supplementary Data 4) encoding a putative PCC, comprising an outer-membrane β-barrel porin, a decaheme *c*-type outer-membrane-associated cytochrome, and two monoheme *c*-type cytochromes predicted to localize to the periplasm and inner membrane, respectively (Fig. 3B).

Structural predictions generated with AlphaFold3, combined with signal peptide and membrane topology analyses (Supplementary Data 5), support a modular electron transfer conduit that spans the inner membrane, periplasm, and outer membrane. The predicted organization (lack of signal peptide) places a monoheme cytochrome anchored in the inner membrane by 4 transmembrane helices, a periplasmic monoheme cytochrome, and an outer-membrane porin associated with a decaheme *c*-type cytochrome extending into the extracellular space (Fig. 3C). This architecture suggests a directional electron transfer pathway from the cytoplasmic membrane toward extracellular electron acceptors. Notably, the second enriched *Methylobacter* MAG (clade A) recovered from the anodic biofilm did not encode this putative PCC-like gene cluster.

### The putative EET gene cluster shows a heterogeneous phylogenetic distribution and conserved synteny across Methylococcales

**Figure 4:**
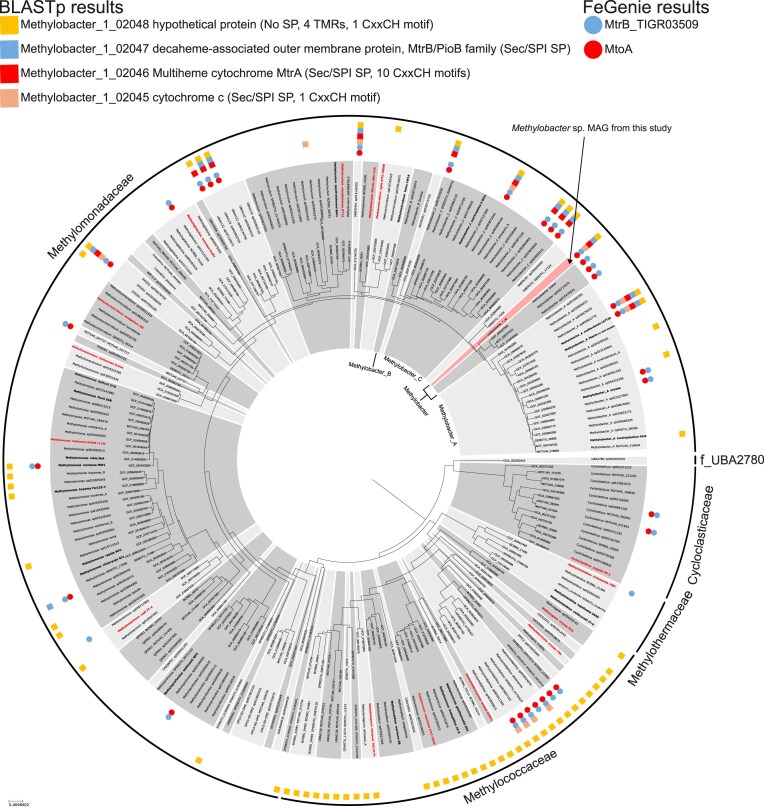
Phylogenetic distribution of the putative EET gene cluster across Methylococcales. Phylogenetic distribution of the putative EET gene cluster across Methylococcales. Maximum-likelihood genome tree constructed from concatenated GTDB-Tk marker genes for species-representative genomes (≥99% CheckM2 completeness). Colored squares indicate high-confidence BLASTp hits (≥90% query coverage, ≥70% amino acid similarity) to the four proteins encoded by the EET gene cluster of the enriched Methylobacter_1 MAG (locus tags 02045–02048). Colored circles denote homologs predicted by FeGenie. Type strains are indicated in bold, with genus type strains shown in red and species type strains in black. Grey background shading denotes genus-level groupings based on GlobDB classification. The Methylobacter_1 MAG from this study is highlighted in red and indicated with an arrow. The scale bar indicates substitutions per site.

The phylogenetic distribution of the putative PCC/EET gene set identified in the enriched *Methylobacter* MAG was evaluated across Methylococcales (Fig. 4). High-confidence BLASTp hits indicated that the complete four-gene set occurred in a limited number of genomes, including SXIZ01 sp026398935, JABFSH01 sp013140705, *Methylobacter_C* sp903873045, *Methylobacter_A* sp903832775, *Methylobacter svalbardensis* (clade A), and the enriched *Methylobacter* MAG (Methylobacter_1).

Across the remaining genomes, partial variants were more common. The combination of outer-membrane porin (Methylobacter_1_02047; MtrB/PioB family), the decaheme *c*-type cytochrome (Methylobacter_1_02046; MtrA family), and the inner-membrane-anchored monoheme *c*-type cytochrome (Methylobacter_1_02048) were detected in *Methylobacter_C* sp903839855, *Methylobacter_C* sp903859415, *Methylobacter_C* sp903863905, *Methylobacter_C* sp903934035, UBA4132 sp02134785, and UBA4132 sp903902705. The periplasmic monoheme c-type cytochrome (Methylobacter_1_02045) was absent from these genomes, whereas the outer-membrane porin, decaheme cytochrome, and inner-membrane monoheme cytochrome were retained. Thus, within Methylococcales, the full gene set appears restricted to a small subset of lineages, while incomplete configurations, most commonly lacking the periplasmic cytochrome component, were distributed more broadly (Fig. 4).

**Figure 5:**
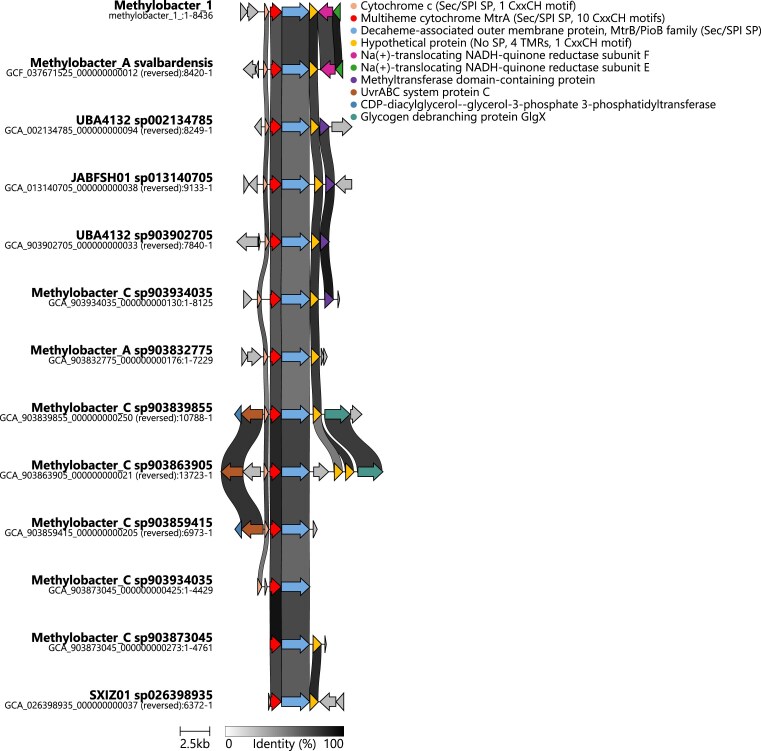
Synteny of the EET locus across representative Methylococcales genomes. Genomic regions spanning the four EET genes and adjacent flanking coding sequences are shown for each genome. Genes are drawn to scale with orientation preserved. Bold headings indicate GTDB-Tk taxonomic assignments, with genome identifiers and locus tags from GlobDB shown below. Gray shading denotes BLASTp identity between homologous genes relative to the genome displayed directly above, with intensity proportional to sequence similarity; shading links only homologous genes between adjacent genomes. The scale bar represents gene length.

To assess structural conservation of the EET locus, genomic regions spanning the four genes were compared across the 11 representative Methylococcales genomes mentioned before. Gene order and orientation were conserved in all genomes containing the locus, and homologues of the outer-membrane porin, decaheme *c*-type cytochrome, and inner-membrane anchored monoheme *c-*type cytochrome were consistently co-localized (Fig. 5). In one genome (Methylobacter_C sp903873045), the locus was split across two contigs, with the periplasmic and membrane-bound cytochrome homologs located separately, consistent with assembly fragmentation. Divergent homologs of the periplasmic monoheme *c*-type cytochrome were detected in most genomes previously classified as incomplete, whereas only SXIZ01 sp026398935 lacked a recognizable homolog within the locus region. Overall, the locus architecture was conserved despite sequence divergence or absence of the periplasmic component.

## Discussion

Here we report the enrichment of aerobic methanotrophic bacteria under anoxic conditions on poised electrodes. We elucidate their potential EET pathways and show that these are present across multiple Methylococcales lineages.

Current generation by biofilms of aerobic methanotrophs has been reported in methane-fed microbial fuel cells operated under oxic conditions (Jawaharraj et al. 2021). Our results extend these observations by demonstrating that CH_4_-dependent electrogenic activity by aerobic methanotrophs can emerge under anoxic conditions in mixed-community biofilms. Unlike previous studies that relied on axenic or highly enriched cultures to demonstrate CH_4_-linked current generation, our system was inoculated with an environmental microcosm enrichment, yet bioelectrochemical operation selectively enriched MOB. This highlights BESs as not only experimental platforms for characterizing known electroactive methanotrophs, but also as selective enrichment tools for uncovering previously unrecognized electroactive CH_4_ oxidizers from complex communities.

Anaerobic CH_4_-dependent EET has been most extensively characterized for the anaerobic methanotrophic archaeon *Ca*. Methanoperedens, which couples CH_4_ oxidation to electron transfer under strictly anoxic conditions and achieves high fractions of CH_4_-dependent current in highly enriched bioanodes. For example, gold-mesh anodes dominated by *Ca*. Methanoperedens (up to 82% relative abundance) produced 91%–93% CH_4_-dependent current with peak current densities of ∼125 mA ∙ m⁻² (Ouboter et al. 2024). The absence of *Ca*. Methanoperedens and other canonical ANME lineages in our anodic enrichment indicate that anaerobic CH_4_-linked electron transfer to electrodes is not restricted to ANME archaea.

In a mixed-community BES, such as the one studied here, CH_4_-dependent current is typically superimposed on background electrogenic activity arising from heterotrophic processes within the biofilm (An Lee 2013, Lóránt et al. 2019). Against this background, the reproducible response to CH_4_ removal and re-addition indicates that CH_4_ oxidation by aerobic methanotrophs directly contributed to electron flux toward the anode under anoxic conditions.

Both genome-resolved read mapping and marker gene-based community profiling indicated selective enrichment of *Methylobacter* spp. on the anode, with no other genera showing comparable increases in relative abundance. However, a substantial fraction of the inoculum-derived taxa remained detectable in the anodic biofilm. Importantly, physical association with the electrode does not imply direct involvement in EET. Micro-organisms may colonize the electrode surface due to spatial proximity to substrates, cross-feeding on metabolites produced by electrogenic partners, or general biofilm-forming capacity. Consequently, the presence of phylogenetically diverse taxa on the anode should not be interpreted as evidence for their direct contribution to electron transfer. Among the enriched *Methylobacter* MAGs, only a single genome encoded a PCC-like conduit, identifying it as the most plausible candidate for CH_4_-linked electrogenic activity.

CH_4_ activation by aerobic methanotrophs requires molecular oxygen; thus, the observation of CH_4_-linked current under bulk anoxic conditions implies that methane mono-oxygenase activity is sustained by trace oxygen availability within the biofilm. However, oxygen measurements were restricted to bulk anolyte concentrations, precluding resolution of the spatial distribution and origin of potential micro-oxic niches within the biofilm.

Genomic features of the enriched *Methylobacter* MAG indicate adaptation to such low-oxygen niches, rather than oxygen-independent CH_4_ activation (Guerrero-Cruz et al. 2021). The genome encoded high-affinity terminal oxidases, including *o*- and *bd*-type quinol oxidases, which are characteristic of methanotrophs inhabiting oxic-anoxic interfaces and enable respiration at very low oxygen concentrations (Borisov et al. 2011, Skennerton et al. 2015). In addition, it encoded a bacteriohemerythrin, a protein proposed to bind and buffer oxygen and thereby stabilize aerobic metabolism under hypoxic and fluctuating oxygen conditions (Rahalkar Bahulikar 2018, Weiblen et al. 2025)

Importantly, these low-oxygen adaptations do not directly explain CH_4_-linked current generation per se, as electrons derived from CH_4_ oxidation would preferentially be consumed by terminal oxidases whenever oxygen is available. Instead, they indicate that the *Methylobacter* MAG is physiologically equipped to sustain CH_4_ oxidation under conditions where oxygen availability is transient and spatially constrained. While some alphaproteobacterial methanotrophs synthesize methanobactin, a copper-binding compound proposed to enable CH_4_ oxidation under anoxic conditions via intracellular oxygen generation from water-splitting (Dershwitz et al. 2021), methanobactin-related genes were not detected in the *Methylobacter* MAG analyzed here. Together with bacteriohemerythrins and high-affinity terminal oxidases, such strategies illustrate that methanotrophs employ multiple, lineage-specific solutions to sustain aerobic CH_4_ oxidation at oxic-anoxic boundaries.

The observation of CH_4_-dependent current indicates that methane oxidation under anoxic conditions can be linked to electron transfer toward an electrode, rather than being solely coupled to aerobic respiration. We hypothesize that *Methylobacter* spp. sustains oxygen-dependent methane activation at trace O₂ availability and redirects electrons toward extracellular acceptors when oxygen is limiting and spatially restricted within the biofilm, potentially reflecting a metabolic allocation strategy in which O₂ is preferentially used for CH_4_ activation rather than as the dominant terminal electron acceptor.

The enriched MAG also encoded pathways for sulfide, thiosulfate, and sulfur oxidation (Sqr, FccAB, SoxXYZABCD). Therefore, electrons derived from the oxidation of reduced sulfur compounds may have contributed to the measured current, representing a potential secondary electron source alongside methane oxidation. However, this contribution is likely limited, as the medium was sulfate-free and sulfate was present only in trace amounts derived from antibiotic counterions, biomass turnover, or residual inoculum from the enrichment culture.

Nitrogen oxide reduction has been reported as an alternative electron sink supporting methane oxidation under oxygen limitation in some methanotrophs; our MAG lacked the genomic hallmarks of respiratory nitrate/nitrite reduction. In contrast, oxygen-limited *Methylobacter* spp. have been reported to couple methane oxidation to nitrite reduction with N₂O production under hypoxic conditions (Hao et al. 2022), and methane oxidation coupled to nitrate reduction has been demonstrated for *Methylomonas denitrificans* under oxygen limitation (Kits et al. 2015, Orata et al. 2018).

In Gram-negative bacteria, EET to insoluble acceptors is mediated by pathways that connect inner-membrane electron carriers, via periplasmic cytochromes, to outer-membrane modules that deliver electrons beyond the cell surface. In the best-characterized electrogenic bacteria, such as *Shewanella oneidensis* MR-1 and *Geobacter sulfurreducens*, this function is achieved through MHC-based systems, including PCCs or alternative outer-membrane cytochrome assemblies, that establish a continuous electron pathway from the cytoplasmic membrane to extracellular electron acceptors (Paquete et al. 2022). In the case of PCCs, this is facilitated by the association of a linear decaheme *c*-type cytochrome with a β-barrel porin, forming a critical bridge across the outer membrane.

The PCC-like module encoded by the enriched *Methylobacter* MAG conforms to this core organizational principle. The presence of a decaheme *c*-type cytochrome of the MtrA/DmsE family associated with an outer-membrane β-barrel of the MtrB/PioB family provides a plausible conduit for electron transfer across the outer membrane, analogous to the central MtrAB unit of the *Shewanella* MtrCAB system. Its linkage to cytochromes, predicted to localize to the periplasm and inner membrane, establishes a potential continuous pathway for electron flow from the cytoplasmic membrane toward extracellular acceptors.

At the same time, the *Methylobacter* PCC lacks several components that characterize canonical electrogenic systems. No homologues of outer-surface MHCs such as MtrC/OmcA in *Shewanella* or OmcB/OmcC in *Geobacter* were detected, nor were genes encoding cytochrome nanowires detected. This suggests that EET in *Methylobacter* may rely on direct interaction of the outer-membrane decaheme cytochrome with insoluble electron acceptors or on close cell-surface contact, rather than on extended EET networks.

However, mediated electron transfer via soluble redox-active compounds cannot be excluded in the present study. Flavins, including riboflavin present in the medium, are known to function as extracellular electron shuttles in electrogenic systems (Liu et al. 2025). In addition, indirect contributions from other biofilm members, like the enriched *Methylobacter* population (clade A; not harboring the putative EET locus), may have occurred via cross-feeding or leakage of reduced metabolites. Because medium replacement experiments and electrochemical characterization of redox-active compounds (scan-rate-dependent cyclic voltammetry) were not performed, the contribution of these mechanisms could not be resolved.

Phylogenomic analysis shows that the gene cluster identified in the enriched *Methylobacter* MAG is present across multiple lineages, yet few genomes within the Methylococcales. Partial variants were more widely distributed, most commonly lacking the periplasmic monoheme *c*-type cytochrome. This pattern indicates that PCC-like systems in Methylococcales are modular and variably assembled, rather than forming a single conserved EET system.

The recurrent co-occurrence of the outer-membrane porin and the decaheme *c*-type cytochrome, and in some cases an associated inner-membrane *c*-type cytochrome, suggests that PCC-like *c*-type cytochrome arrangements spanning the inner membrane, periplasm, and outer membrane occur across the order, even in the absence of the complete gene cluster. The inconsistent presence of the periplasmic monoheme *c*-type cytochrome indicates that this component is not universally retained within these configurations. Taken together, these phylogenomic patterns suggest that the complete gene cluster identified in our MAG represents a specific configuration within a broader spectrum of *c*-type cytochrome-porin arrangements in Methylococcales, underscoring heterogeneity in gene content and organization without implying conserved function.

Consistent with this interpretation, comparative synteny analysis revealed that genomes harboring the locus retain a conserved gene order and orientation across the region, despite being dispersed across the phylogeny. Such conservation of local operon structure combined with a scattered taxonomic distribution is consistent with horizontal gene transfer of the EET locus rather than strict vertical inheritance. In contrast, the periplasmic monoheme *c*-type cytochrome exhibited greater sequence divergence and, in at least one lineage, apparent absence, further suggesting flexibility in this position while maintaining the core porin-decaheme architecture.

In the Bothnian Sea, CH_4_ oxidation below the sulfate-methane transition zone has been attributed to anaerobic processes, with geochemical profiles and incubation experiments supporting coupling of CH_4_ oxidation to iron reduction in strictly anoxic sediments (Egger et al. 2015). Metagenomic analyses from the same system detected members of the Methylococcales, including *Methylobacter*-affiliated sequences, predominantly in sediment horizons where CH_4_ co-occurred with oxygen, consistent with the requirement of oxygen for aerobic methanotrophy (Rasigraf et al. 2017). The presence of these taxa was interpreted as reflecting CH_4_ oxidation at oxic-anoxic transition zones rather than activity in fully anoxic sediment.

In the *in situ* sediment, *Methylobacter* was present only at trace abundance, and the lineage corresponding to the anode-derived MAG was not detected. Our enrichment demonstrates that sediment-derived microcosms can yield *Methylobacter* populations capable of sustaining CH_4_-dependent electron transfer under anoxic conditions when a solid electron acceptor in the form of a poised anode is provided. This suggests that aerobic methanotrophs detected at oxic-anoxic interfaces may persist under conditions of extreme oxygen limitation, potentially sustaining CH_4_ oxidation during transient oxygen exposure. Rather than contradicting established anaerobic CH_4_ oxidation pathways, such behavior may reflect niche partitioning at redox interfaces, where aerobic methanotrophs and ANME occupy adjacent but distinct microenvironments shaped by chemical gradients.

Several limitations constrain the mechanistic interpretation of CH_4_-dependent current generation in our system. First, current reflects net electron flux to the anode and does not resolve the relative contributions of CH_4_ oxidation, sulfur oxidation, endogenous decay, or cross-feeding processes within the biofilm. Nevertheless, the rapid and reproducible decrease in current upon CH_4_ removal, and its recovery upon CH_4_ re-addition, indicates that methane availability directly controls a substantial fraction of the anodic electron flux, rendering alternative processes unlikely to explain the observed dynamics. Second, CH_4_ activation by aerobic methanotrophs requires oxygen, yet oxygen measurements were limited to bulk anolyte concentrations, and the presence of micro-oxic niches within the biofilm could not be excluded. Third, the proposed PCC-like conduit and associated *c*-type cytochromes were inferred from gene content and structural prediction, and gene presence does not establish expression, directionality of electron flow, or coupling to electrode reduction. Fourth, the operation of a poised-anode BES imposes a strong selective pressure, such that enrichment under these conditions does not reflect the *in situ* abundance or activity of electrogenic methanotrophs in coastal sediments. Incubation at room temperature constitutes an additional selective factor; although this was consistent with the original sediment pre-incubation and subsequent enrichment by Klomp et al. (2026), it likely favored mesophilic over psychrophilic methanotrophs and may have excluded taxa active at lower temperatures in the native environment. Consequently, the enriched community should not be interpreted as representative of dominant methane-oxidizing populations *in situ*. Finally, the phylogenomic survey identifies homologous gene configurations across Methylococcales, but does not establish whether these modules are functionally equivalent, support growth, or mediate EET under environmentally relevant conditions.

A key next step will be to directly link CH_4_ oxidation to electron discharge and carbon assimilation under controlled conditions. Experiments with pure cultures supplied with labeled CH_4_ would allow closure of both the carbon and electron balance, enabling assessment of coulombic efficiency and the fraction of CH_4_-derived electrons recovered at the electrode. Such measurements would clarify whether EET can support biomass formation in aerobic methanotrophs or whether it primarily represents a survival strategy that sustains minimal energy conservation under oxygen limitation. Transcriptomic and proteomic analyses under electrogenic conditions will be essential to determine whether the putative EET module is actively expressed and translated during CH_4_-dependent current generation, thereby moving from genomic potential toward functional validation.

## Conclusion

In summary, an anoxic, poised-anode BES inoculated with a coastal sediment enrichment produced reproducible CH_4_-dependent current and selectively enriched *Methylobacter* populations on the anode. Genome-resolved analyses identified a PCC-like cytochrome-porin module and an expanded cytochrome repertoire in the most likely CH_4_-linked electrogenic candidate, and phylogenomics showed that related configurations occur across multiple Methylococcales lineages, albeit rarely as a complete gene set observed in our MAG. Comparative synteny further demonstrated conserved locus organization across genomes, consistent with horizontal transfer of this EET module. Together, these findings support the hypothesis that aerobic methanotrophs can contribute to electron transfer toward extracellular acceptors under anoxic conditions when CH_4_ is available, and they provide a framework for testing whether such interactions influence CH_4_ turnover at oxic-anoxic boundaries in coastal sediments.

## Supplementary Material

fiag067_Supplemental_Files

## Data Availability

Raw sequencing data generated in this study have been deposited in the European Nucleotide Archive under BioProject accession PRJEB108241 (Extracellular Electron Transfer of a *Methylobacter* Enrichment). MAGs meeting medium- and high-quality standards that were taxonomically classified have been deposited under the same BioProject. Relevant accessions include ERS29752532 (inoculum *Methylobacter* clade A MAG), ERS29752579 (anode *Methylobacter* clade A MAG), and ERS29752583 (EET-encoding *Methylobacter* MAG described in this study). To support reproducibility of genome-resolved analyses, including read mapping with CoverM, the complete set of recovered bins used as the mapping reference, including low-quality, unclassified bins and the bin_info output from aviary, is available at *https://doi.org/10.6084/m9.figshare.32134456*. Amino acid FASTA sequences of the Methylobacter_1 bin are also provided via the same repository.
