# Supplementary material for: Electrogenic CH4 oxidation on a bioanode: putative extracellular electron transport system in Methylobacter sp
Source: FEMS Microbiol Ecol. 2026 Jun 23;102(7):fiag067. doi: 10.1093/femsec/fiag067 (PMC13322032; doi:10.1093/femsec/fiag067)
Supplement: fiag067_Supplemental_Files [file fiag067_supplemental_files.zip › Suppl_Data4.docx]

>Methylobacter_1_02045 Cytochrome c (Sec/SPI SP, 1 CxxCH motif)

MKPFYSLHLQTFSSFALALFCFSANAEAVDADAAKAFAKENTCLRCHGVSKDKDGPAFTK

IAAKFKDAPNAEEKLLHHLSSGETAKFPDGHEEPHKILKSDDADAIKNLVDWILSL

>Methylobacter_1_02046 Multiheme cytochrome MtrA (Sec/SPI SP, 10 CxxCH motifs)

MNILITIIMLGGLIMGSAQAEINLPTLGSPTAKASTEVVDKDAVCTRCHDESETKPILAI

YQTPHGNRADPRTPSCQSCHGESEKHLSGASTEESRPASDVVFGTKRGTSGTEPSEAKDQ

NTACLSCHEKDPKRTHWQGGAHQVNDVACTSCHITHAAHDKVRDKKTQPDVCFTCHQEQR

AQTHKFSHHPITEGKVACSDCHNPHGSAGPKLLVKNTVNETCFTCHAEKRGPMLWEHQPV

TEDCTLCHTPHGSNITPLLNSRTPFLCSECHDGPHHSKDPAAGSATGIQGGLTAVPSENY

TGRACLNCHSMVHGSNHPAGALLHR

>Methylobacter_1_02047 Decaheme-associated outer membrane protein, MtrB/PioB family (Sec/SPI SP)

MNTDNEKMKVNVLALAVRCALMAAFAMPLSAQAEDEDEAVALKHPSNTVEFGALYVSQQS

AKFGQYSGLEGQGFYGLGGFDIRGGKGYDGKDSALRWQLNGSNLGTTARTLGGSISEQGK

WKFSLGYDELRHNITDTYQTPLQGETGGNTFNLPDDFGTINAGSSAPSARALNPTQLNAF

HREKEYTTRRNVPISASYFFSPEFSAQVDFNHLEQSGAKLIGTGSQGGINLLGGSTGRAE

AVNIIMNPTQYTTESINAVLNWTGDKAHLSGGYYGSLFHNDYNSLSWQNALASGASACSG

PDCYVNNTMSTAPSNTLHQANLNGGYAFTPTTKLAGGFSYGYNKQDDSFAPTLIPQANGT

PFDMMQPGGLPVSSLNAHVETKHADLKLTNQSIKDLTLTAGFKYNERDNRTPSYTYLYHN

IGGADYTGVNTPYSNRKTQYEVAADYRLTKNQKLRLAYDRDHTRRWCNGVAGGAECVASP

SSIEDKIGLTYRLKAFEDVNFNAGYSYATRNADFSQLYLANTGSYGGEINSQNKLSFVAY

PYNSRQQHVMKTGVNWQATQKLDLGVNGRFSHDNYDATLGVQNGHSAAVNVDATYSYTEN

NSISAYWSWQTQERDLRSGNNGSPTLAPTNIWTNQLTNYSNAVGLLTRHGGLLGGKLEII

GDLSYALDTTSYSTQVPYDPTCVDAGKLTCGTLPAIKNELLSFKLTGNYQVHKNGKVSLA

YIYQKLNSSDYYYNGQAFGFTPNRVMPTGLREQDYTVNVVALSYLYNF

>Methylobacter_1_02048 Hypothetical protein (No SP, 4 TMRs, 1 CxxCH motif)

MIEPKDDLTLGKLSLTYRALFTGFLLVMGLGLLMAGAQIMLTHGLADGKPGLSMNDIVYS

YYGNRSGSKLEAALTGSMKAKAPEAVKFTLIQWVRDGAPDAEWGKIGPLLEKHCASCHDE

ESGLPEVAKQEVAKSLAEIDHGASIASLTRVSHIHLFGIGFIFLFVGWIFGMAEFNQFWK

LILISTPFAFLIIDVASWWLTKFWPGFAWLTMIGGLGYSLASTVMFATSLAQMWLPRYTR

Q
