# Supplementary material for: Electrogenic CH4 oxidation on a bioanode: putative extracellular electron transport system in Methylobacter sp
Source: FEMS Microbiol Ecol. 2026 Jun 23;102(7):fiag067. doi: 10.1093/femsec/fiag067 (PMC13322032; doi:10.1093/femsec/fiag067)
Supplement: fiag067_Supplemental_Files [file fiag067_supplemental_files.zip › Suppl_Data5.docx]

---

DeepTMHMM gff-version 3 output

# Methylobacter_1_02045 Length: 116

# Methylobacter_1_02045 Number of predicted TMRs: 0

Methylobacter_1_02045 signal 1 28

Methylobacter_1_02045 outside 29 116

//

# Methylobacter_1_02046 Length: 325

# Methylobacter_1_02046 Number of predicted TMRs: 0

Methylobacter_1_02046 signal 1 20

Methylobacter_1_02046 outside 21 325

//

# Methylobacter_1_02047 Length: 768

# Methylobacter_1_02047 Number of predicted TMRs: 26

Methylobacter_1_02047 signal 1 33

Methylobacter_1_02047 periplasm 34 46

Methylobacter_1_02047 Beta sheet 47 55

Methylobacter_1_02047 outside 56 73

Methylobacter_1_02047 Beta sheet 74 84

Methylobacter_1_02047 periplasm 85 94

Methylobacter_1_02047 Beta sheet 95 104

Methylobacter_1_02047 outside 105 105

Methylobacter_1_02047 Beta sheet 106 114

Methylobacter_1_02047 periplasm 115 122

Methylobacter_1_02047 Beta sheet 123 130

Methylobacter_1_02047 outside 131 189

Methylobacter_1_02047 Beta sheet 190 197

Methylobacter_1_02047 periplasm 198 205

Methylobacter_1_02047 Beta sheet 206 213

Methylobacter_1_02047 outside 214 253

Methylobacter_1_02047 Beta sheet 254 262

Methylobacter_1_02047 periplasm 263 267

Methylobacter_1_02047 Beta sheet 268 276

Methylobacter_1_02047 outside 277 317

Methylobacter_1_02047 Beta sheet 318 326

Methylobacter_1_02047 periplasm 327 333

Methylobacter_1_02047 Beta sheet 334 341

Methylobacter_1_02047 outside 342 382

Methylobacter_1_02047 Beta sheet 383 390

Methylobacter_1_02047 periplasm 391 397

Methylobacter_1_02047 Beta sheet 398 404

Methylobacter_1_02047 outside 405 438

Methylobacter_1_02047 Beta sheet 439 446

Methylobacter_1_02047 periplasm 447 452

Methylobacter_1_02047 Beta sheet 453 460

Methylobacter_1_02047 outside 461 485

Methylobacter_1_02047 Beta sheet 486 493

Methylobacter_1_02047 periplasm 494 500

Methylobacter_1_02047 Beta sheet 501 508

Methylobacter_1_02047 outside 509 547

Methylobacter_1_02047 Beta sheet 548 556

Methylobacter_1_02047 periplasm 557 562

Methylobacter_1_02047 Beta sheet 563 571

Methylobacter_1_02047 outside 572 585

Methylobacter_1_02047 Beta sheet 586 595

Methylobacter_1_02047 periplasm 596 601

Methylobacter_1_02047 Beta sheet 602 610

Methylobacter_1_02047 outside 611 640

Methylobacter_1_02047 Beta sheet 641 649

Methylobacter_1_02047 periplasm 650 657

Methylobacter_1_02047 Beta sheet 658 666

Methylobacter_1_02047 outside 667 700

Methylobacter_1_02047 Beta sheet 701 709

Methylobacter_1_02047 periplasm 710 715

Methylobacter_1_02047 Beta sheet 716 724

Methylobacter_1_02047 outside 725 757

Methylobacter_1_02047 Beta sheet 758 766

Methylobacter_1_02047 periplasm 767 768

//

# Methylobacter_1_02048 Length: 241

# Methylobacter_1_02048 Number of predicted TMRs: 4

Methylobacter_1_02048 inside 1 18

Methylobacter_1_02048 TMhelix 19 37

Methylobacter_1_02048 outside 38 150

Methylobacter_1_02048 TMhelix 151 171

Methylobacter_1_02048 inside 172 177

Methylobacter_1_02048 TMhelix 178 200

Methylobacter_1_02048 outside 201 206

Methylobacter_1_02048 TMhelix 207 231

Methylobacter_1_02048 inside 232 241

---

# SignalP-6.0 Organism: Other Timestamp: 20250912065814

# ID Prediction OTHER SP(Sec/SPI) LIPO(Sec/SPII) TAT(Tat/SPI) TATLIPO(Tat/SPII) PILIN(Sec/SPIII) CS Position

Methylobacter_1_02045 Cytochrome c _Sec_SPI SP, 1 CxxCH motif_ SP 0.000254 0.999008 0.000218 0.000220 0.000171 0.000170 CS pos: 28-29. Pr: 0.5711

Methylobacter_1_02046 Multiheme cytochrome MtrA _Sec_SPI SP, 10 CxxCH motifs_ SP 0.000185 0.999211 0.000163 0.000168 0.000134 0.000137 CS pos: 20-21. Pr: 0.9782

Methylobacter_1_02047 Decaheme-associated outer membrane protein, MtrB_PioB family _Sec_SPI SP_ SP 0.004816 0.988651 0.005155 0.000536 0.000402 0.000427 CS pos: 33-34. Pr: 0.9731

Methylobacter_1_02048 Hypothetical protein _No SP, 4 TMRs, 1 CxxCH motif_ OTHER 0.999985 0.000000 0.000000 0.000000 0.000000 0.000000
